# 3-Methyl-4-{[(3-{[(3-methyl-5-oxo-1-phenyl-4,5-dihydro-1*H*-pyrazol-4-yl­idene)(phen­yl)meth­yl]amino­meth­yl}benz­yl)amino](phen­yl)methyl­idene}-1-phenyl-1*H*-pyrazol-5(4*H*)-one

**DOI:** 10.1107/S1600536811027000

**Published:** 2011-07-13

**Authors:** Hong-Xin Cai, Wei-Na Wu, Xiao-Xia Li, Yuan Wang

**Affiliations:** a Department of Physics and Chemistry, Henan Polytechnic University, Jiaozuo 454000, People’s Republic of China; b Institute of Functional Materials, Jiangxi University of Finance & Economics, Nanchang 330013, People’s Republic of China

## Abstract

The complete mol­ecule of the title compound, C_42_H_36_N_6_O_2_, is generated by a crystallographic twofold axis with two C atoms of the central phenyl group lying on the axis. In the independent part of the mol­ecule, one amino group is involved in an intra­molecular N—H⋯O hydrogen bond, and the two adjacent phenyl rings are twisted from the plane of the pyrazolone ring with dihedral angles of 6.82 (3) and 88.32 (6)°. The crystal packing exhibits no classical inter­molecular contacts.

## Related literature

For the similar structure (*E*,*E*)-3,3′-dimethyl-1,1′-diphenyl-4,4′-{(3-aza­pentane-1,5-diyldiimino)­bis­[phenyl­methyl­idyne]}di-1*H*-pyrazol-5(4*H*)-one, see: Zhang *et al.* (2010 ▶). For the DNA binding properties of transition metal complexes with the above Schiff base, see: Wang & Yang (2005 ▶).
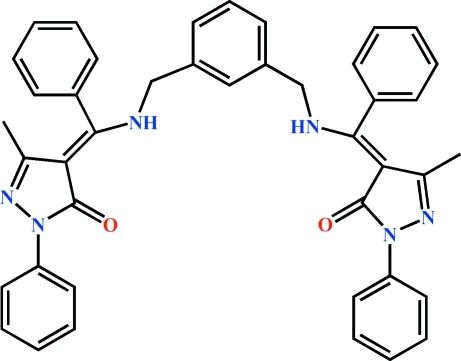


## Experimental

### 

#### Crystal data

C_42_H_36_N_6_O_2_
*M* *_r_* = 656.77
Monoclinic, 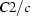

*a* = 26.4648 (5) Å
*b* = 14.3131 (3) Å
*c* = 9.5492 (2) Å
β = 96.766 (1)°
*V* = 3591.98 (13) Å^3^
*Z* = 4
Mo *K*α radiation
μ = 0.08 mm^−1^
*T* = 296 K
0.26 × 0.21 × 0.18 mm

#### Data collection

Bruker APEXII CCD diffractometer
Absorption correction: multi-scan (*SADABS*; Bruker, 2007 ▶) *T*_min_ = 0.981, *T*_max_ = 0.986
26618 measured reflections
4290 independent reflections
2244 reflections with *I* > 2σ(*I*)
*R*_int_ = 0.034

#### Refinement

*R*[*F* ^2^ > 2σ(*F* ^2^)] = 0.049
*wR*(*F* ^2^) = 0.154
*S* = 1.01
4290 reflections
229 parameters
H-atom parameters constrained
Δρ_max_ = 0.14 e Å^−3^
Δρ_min_ = −0.14 e Å^−3^

### 

Data collection: *APEX2* (Bruker, 2007 ▶); cell refinement: *SAINT* (Bruker, 2007 ▶); data reduction: *SAINT*; program(s) used to solve structure: *SHELXS97* (Sheldrick, 2008 ▶); program(s) used to refine structure: *SHELXL97* (Sheldrick, 2008 ▶); molecular graphics: *SHELXTL* (Sheldrick, 2008 ▶); software used to prepare material for publication: *SHELXTL*.

## Supplementary Material

Crystal structure: contains datablock(s) I, global. DOI: 10.1107/S1600536811027000/vm2108sup1.cif

Structure factors: contains datablock(s) I. DOI: 10.1107/S1600536811027000/vm2108Isup2.hkl

Supplementary material file. DOI: 10.1107/S1600536811027000/vm2108Isup3.cml

Additional supplementary materials:  crystallographic information; 3D view; checkCIF report

## Figures and Tables

**Table 1:** Hydrogen-bond geometry (Å, °)

*D*—H⋯*A*	*D*—H	H⋯*A*	*D*⋯*A*	*D*—H⋯*A*
N3—H3*A*⋯O1	0.86	2.04	2.7353 (17)	138

**Fig. 1.**
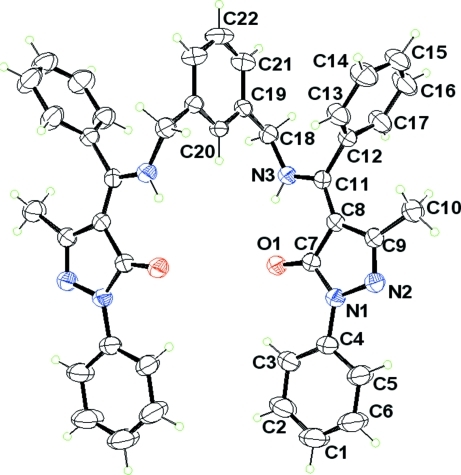
The molecular structure shown with 30% probability displacement ellipsoids. Unlabelled atoms are related with the labelled ones by symmetry operation -x, y, -z + 1/2.

## supplementary crystallographic information

### Comment

For our interest in coordination chemistry of the Schiff bases derived from 1-phenyl-3-methyl-4-benzoyl-5-pyrazolone (PMBP) (Wang *et al.*, 2005; Zhang *et al.*, 2010), the crystal structure of the title compound was determined by X-ray diffraction analysis.

As shown in Fig.1, the molecule is generated by a crystallographic two fold axis, with atoms C20 and C22 lying on the axis. In the independent part of the molecule, the two phenyl rings (C1—C6, r.m.s. deviation = 0.0023 Å and C12—C17, r.m.s. deviation = 0.0060 Å) are slightly twisted with respect to the central pyrazolone ring (r.m.s. deviation = 0.0040 Å) making dihedral angles of 6.82 (3)° and 88.32 (6)°, respectively. The non-hydrogen atoms of the phenylenedimethylene group are situated in a fair plane (r.m.s. deviation = 0.0013 Å). The dihedral angles between this plane and the two phenyls in the PMBP moiety are 36.27 (8)° and 82.61 (6)°, respectively. The conformation of the molecule is influenced by an intramolecular hydrogen bond N3—H3A···O1 (Table 1).

### Experimental

A quality of PMBP (1.1 g, 4 mmol) was dissolved in EtOH (50 ml), and an EtOH solution (10 ml) containing (3-(aminomethyl)phenyl)methanamine (0.3 g, 2 mmol) was added dropwise. The mixture was refluxed on a water bath for 3 h, then cooled to room temperature. Yellow block crystals were obtained by slow evaporation of the reaction mixture.

### Refinement

All H atoms were placed in calculated positions, with the carrier atom-H distances = 0.93 Å for aryl, 0.97 Å for methylene, 0.96 Å for the methyl and 0.86 Å for the secondary amine H atoms, and refined as riding, with the *U*_iso_(H) = 1.5Ueq(C) for methyl groups and 1.2Ueq(C,*N*) for others.

### Crystal data

**Table:**

C_42_H_36_N_6_O_2_	*F*(000) = 1384
*M**_r_* = 656.77	*D*_x_ = 1.214 Mg m^−^^3^
Monoclinic, *C*2/*c*	Mo *K*α radiation, λ = 0.71073 Å
Hall symbol: -C 2yc	Cell parameters from 5169 reflections
*a* = 26.4648 (5) Å	θ = 2.5–22.3°
*b* = 14.3131 (3) Å	µ = 0.08 mm^−^^1^
*c* = 9.5492 (2) Å	*T* = 296 K
β = 96.766 (1)°	Block, yellow
*V* = 3591.98 (13) Å^3^	0.26 × 0.21 × 0.18 mm
*Z* = 4	

### Data collection

**Table:**

Bruker APEXII CCD diffractometer	4290 independent reflections
Radiation source: fine-focus sealed tube	2244 reflections with *I* > 2σ(*I*)
graphite	*R*_int_ = 0.034
φ and ω scans	θ_max_ = 27.9°, θ_min_ = 1.6°
Absorption correction: multi-scan (*SADABS*; Bruker, 2007)	*h* = −34→34
*T*_min_ = 0.981, *T*_max_ = 0.986	*k* = −18→18
26618 measured reflections	*l* = −12→12

### Refinement

**Table:**

Refinement on *F*^2^	Secondary atom site location: difference Fourier map
Least-squares matrix: full	Hydrogen site location: inferred from neighbouring sites
*R*[*F*^2^ > 2σ(*F*^2^)] = 0.049	H-atom parameters constrained
*wR*(*F*^2^) = 0.154	*w* = 1/[σ^2^(*F*_o_^2^) + (0.070*P*)^2^ + 0.8006*P*] where *P* = (*F*_o_^2^ + 2*F*_c_^2^)/3
*S* = 1.01	(Δ/σ)_max_ < 0.001
4290 reflections	Δρ_max_ = 0.14 e Å^−^^3^
229 parameters	Δρ_min_ = −0.14 e Å^−^^3^
0 restraints	Extinction correction: *SHELXL97* (Sheldrick, 2008), Fc^*^=kFc[1+0.001xFc^2^λ^3^/sin(2θ)]^-1/4^
Primary atom site location: structure-invariant direct methods	Extinction coefficient: 0.0014 (3)

### Special details

**Table:**

Geometry. All e.s.d.'s (except the e.s.d. in the dihedral angle between two l.s. planes) are estimated using the full covariance matrix. The cell e.s.d.'s are taken into account individually in the estimation of e.s.d.'s in distances, angles and torsion angles; correlations between e.s.d.'s in cell parameters are only used when they are defined by crystal symmetry. An approximate (isotropic) treatment of cell e.s.d.'s is used for estimating e.s.d.'s involving l.s. planes.
Refinement. Refinement of *F*^2^ against ALL reflections. The weighted *R*-factor *wR* and goodness of fit *S* are based on *F*^2^, conventional *R*-factors *R* are based on *F*, with *F* set to zero for negative *F*^2^. The threshold expression of *F*^2^ > σ(*F*^2^) is used only for calculating *R*-factors(gt) *etc*. and is not relevant to the choice of reflections for refinement. *R*-factors based on *F*^2^ are statistically about twice as large as those based on *F*, and *R*- factors based on ALL data will be even larger.

### Fractional atomic coordinates and isotropic or equivalent isotropic displacement parameters (Å^2^)

**Table:**

	*x*	*y*	*z*	*U*_iso_*/*U*_eq_	
C1	0.10591 (12)	0.05358 (17)	0.2444 (3)	0.1384 (12)	
H1	0.1008	−0.0107	0.2429	0.166*	
C2	0.06985 (11)	0.11231 (16)	0.1809 (3)	0.1245 (10)	
H2	0.0399	0.0873	0.1351	0.149*	
C3	0.07646 (8)	0.20769 (14)	0.1825 (3)	0.0910 (7)	
H3	0.0513	0.2466	0.1386	0.109*	
C4	0.12058 (7)	0.24467 (12)	0.2496 (2)	0.0697 (5)	
C5	0.15709 (9)	0.18561 (15)	0.3125 (3)	0.1080 (9)	
H5	0.1874	0.2098	0.3576	0.130*	
C6	0.14915 (12)	0.09075 (16)	0.3094 (4)	0.1417 (13)	
H6	0.1742	0.0514	0.3531	0.170*	
C7	0.10172 (6)	0.41413 (11)	0.18660 (19)	0.0584 (4)	
C8	0.12958 (6)	0.49712 (11)	0.23182 (18)	0.0561 (4)	
C9	0.17273 (6)	0.46540 (13)	0.32301 (18)	0.0625 (5)	
C10	0.21466 (8)	0.51993 (14)	0.4038 (2)	0.0895 (7)	
H10A	0.2379	0.4778	0.4564	0.134*	
H10B	0.2006	0.5618	0.4675	0.134*	
H10C	0.2324	0.5552	0.3394	0.134*	
C11	0.11435 (6)	0.58586 (11)	0.18703 (18)	0.0551 (4)	
C12	0.14287 (6)	0.67116 (11)	0.23827 (18)	0.0569 (4)	
C13	0.13372 (8)	0.71405 (13)	0.3608 (2)	0.0756 (5)	
H13	0.1088	0.6908	0.4124	0.091*	
C14	0.16157 (9)	0.79207 (16)	0.4079 (2)	0.0951 (7)	
H14	0.1550	0.8216	0.4906	0.114*	
C15	0.19853 (9)	0.82590 (15)	0.3338 (3)	0.0962 (7)	
H15	0.2178	0.8773	0.3674	0.115*	
C16	0.20722 (9)	0.78489 (16)	0.2119 (3)	0.1021 (8)	
H16	0.2319	0.8090	0.1604	0.122*	
C17	0.17952 (8)	0.70699 (14)	0.1631 (2)	0.0858 (6)	
H17	0.1858	0.6789	0.0790	0.103*	
C18	0.05088 (7)	0.68359 (12)	0.0429 (2)	0.0706 (5)	
H18A	0.0265	0.6715	−0.0392	0.085*	
H18B	0.0773	0.7234	0.0133	0.085*	
C19	0.02445 (6)	0.73496 (11)	0.1510 (2)	0.0646 (5)	
C20	0.0000	0.68840 (15)	0.2500	0.0621 (7)	
H20	0.0000	0.6234	0.2500	0.075*	
C21	0.02423 (8)	0.83127 (13)	0.1519 (3)	0.0908 (7)	
H21	0.0405	0.8642	0.0861	0.109*	
C22	0.0000	0.8787 (2)	0.2500	0.1146 (13)	
H22	0.0000	0.9436	0.2500	0.138*	
N1	0.12954 (5)	0.34190 (9)	0.25329 (16)	0.0644 (4)	
N2	0.17324 (5)	0.37498 (10)	0.33522 (16)	0.0694 (4)	
N3	0.07349 (5)	0.59561 (9)	0.09345 (16)	0.0668 (4)	
H3A	0.0591	0.5453	0.0597	0.100*	
O1	0.06148 (4)	0.40607 (8)	0.10539 (14)	0.0743 (4)	

### Atomic displacement parameters (Å^2^)

**Table:**

	*U*^11^	*U*^22^	*U*^33^	*U*^12^	*U*^13^	*U*^23^
C1	0.136 (2)	0.0553 (14)	0.208 (3)	−0.0138 (15)	−0.047 (2)	0.0128 (17)
C2	0.1086 (19)	0.0678 (14)	0.184 (3)	−0.0293 (14)	−0.0386 (19)	0.0169 (16)
C3	0.0764 (13)	0.0606 (12)	0.1302 (19)	−0.0125 (10)	−0.0116 (13)	0.0129 (12)
C4	0.0711 (12)	0.0493 (10)	0.0868 (13)	−0.0025 (9)	0.0016 (10)	−0.0035 (9)
C5	0.0992 (16)	0.0550 (12)	0.156 (2)	0.0067 (11)	−0.0417 (16)	−0.0062 (13)
C6	0.136 (2)	0.0555 (14)	0.215 (3)	0.0057 (14)	−0.062 (2)	0.0043 (16)
C7	0.0522 (9)	0.0513 (10)	0.0712 (11)	−0.0004 (8)	0.0047 (9)	−0.0067 (8)
C8	0.0486 (9)	0.0505 (9)	0.0688 (11)	−0.0022 (7)	0.0048 (8)	−0.0063 (8)
C9	0.0545 (10)	0.0552 (10)	0.0758 (12)	−0.0009 (8)	−0.0003 (9)	−0.0053 (9)
C10	0.0726 (12)	0.0717 (13)	0.1153 (17)	−0.0063 (10)	−0.0257 (12)	−0.0031 (12)
C11	0.0451 (8)	0.0531 (10)	0.0672 (11)	−0.0003 (7)	0.0075 (8)	−0.0058 (8)
C12	0.0517 (9)	0.0484 (9)	0.0690 (11)	−0.0031 (7)	0.0004 (8)	−0.0032 (8)
C13	0.0790 (12)	0.0730 (13)	0.0753 (13)	−0.0160 (10)	0.0109 (10)	−0.0137 (10)
C14	0.1101 (18)	0.0861 (15)	0.0869 (15)	−0.0199 (14)	0.0023 (14)	−0.0292 (12)
C15	0.0974 (16)	0.0703 (14)	0.1161 (19)	−0.0291 (12)	−0.0069 (15)	−0.0165 (14)
C16	0.0932 (16)	0.0910 (16)	0.124 (2)	−0.0426 (13)	0.0228 (15)	−0.0114 (15)
C17	0.0831 (13)	0.0806 (14)	0.0974 (15)	−0.0274 (11)	0.0265 (12)	−0.0194 (12)
C18	0.0580 (10)	0.0597 (11)	0.0907 (14)	−0.0013 (8)	−0.0054 (10)	0.0099 (10)
C19	0.0492 (9)	0.0456 (9)	0.0944 (14)	−0.0025 (7)	−0.0106 (9)	0.0049 (9)
C20	0.0508 (13)	0.0358 (11)	0.0960 (19)	0.000	−0.0072 (13)	0.000
C21	0.0942 (15)	0.0489 (11)	0.130 (2)	−0.0071 (10)	0.0134 (14)	0.0107 (11)
C22	0.131 (3)	0.0378 (14)	0.179 (4)	0.000	0.037 (3)	0.000
N1	0.0574 (8)	0.0501 (8)	0.0826 (10)	−0.0003 (7)	−0.0051 (8)	−0.0051 (7)
N2	0.0603 (9)	0.0580 (9)	0.0862 (11)	0.0006 (7)	−0.0066 (8)	−0.0040 (8)
N3	0.0537 (8)	0.0528 (8)	0.0905 (11)	−0.0027 (6)	−0.0050 (8)	−0.0051 (7)
O1	0.0579 (7)	0.0572 (7)	0.1023 (10)	−0.0039 (6)	−0.0132 (7)	−0.0077 (6)

### Geometric parameters (Å, °)

**Table:**

C1—C6	1.346 (4)	C12—C17	1.372 (2)
C1—C2	1.359 (3)	C13—C14	1.384 (3)
C1—H1	0.9300	C13—H13	0.9300
C2—C3	1.376 (3)	C14—C15	1.362 (3)
C2—H2	0.9300	C14—H14	0.9300
C3—C4	1.370 (3)	C15—C16	1.347 (3)
C3—H3	0.9300	C15—H15	0.9300
C4—C5	1.369 (3)	C16—C17	1.385 (3)
C4—N1	1.412 (2)	C16—H16	0.9300
C5—C6	1.374 (3)	C17—H17	0.9300
C5—H5	0.9300	C18—N3	1.452 (2)
C6—H6	0.9300	C18—C19	1.506 (3)
C7—O1	1.2465 (19)	C18—H18A	0.9700
C7—N1	1.381 (2)	C18—H18B	0.9700
C7—C8	1.437 (2)	C19—C20	1.378 (2)
C8—C11	1.385 (2)	C19—C21	1.379 (2)
C8—C9	1.426 (2)	C20—C19^i^	1.378 (2)
C9—N2	1.299 (2)	C20—H20	0.9300
C9—C10	1.495 (2)	C21—C22	1.375 (2)
C10—H10A	0.9600	C21—H21	0.9300
C10—H10B	0.9600	C22—C21^i^	1.375 (2)
C10—H10C	0.9600	C22—H22	0.9300
C11—N3	1.327 (2)	N1—N2	1.4002 (19)
C11—C12	1.488 (2)	N3—H3A	0.8600
C12—C13	1.368 (2)		
			
C6—C1—C2	118.4 (2)	C12—C13—H13	120.0
C6—C1—H1	120.8	C14—C13—H13	120.0
C2—C1—H1	120.8	C15—C14—C13	120.4 (2)
C1—C2—C3	121.8 (2)	C15—C14—H14	119.8
C1—C2—H2	119.1	C13—C14—H14	119.8
C3—C2—H2	119.1	C16—C15—C14	120.0 (2)
C4—C3—C2	119.2 (2)	C16—C15—H15	120.0
C4—C3—H3	120.4	C14—C15—H15	120.0
C2—C3—H3	120.4	C15—C16—C17	120.3 (2)
C5—C4—C3	119.04 (18)	C15—C16—H16	119.9
C5—C4—N1	119.34 (17)	C17—C16—H16	119.9
C3—C4—N1	121.61 (17)	C12—C17—C16	120.2 (2)
C4—C5—C6	120.2 (2)	C12—C17—H17	119.9
C4—C5—H5	119.9	C16—C17—H17	119.9
C6—C5—H5	119.9	N3—C18—C19	113.69 (15)
C1—C6—C5	121.4 (2)	N3—C18—H18A	108.8
C1—C6—H6	119.3	C19—C18—H18A	108.8
C5—C6—H6	119.3	N3—C18—H18B	108.8
O1—C7—N1	126.00 (15)	C19—C18—H18B	108.8
O1—C7—C8	129.32 (15)	H18A—C18—H18B	107.7
N1—C7—C8	104.68 (14)	C20—C19—C21	118.47 (19)
C11—C8—C9	131.56 (15)	C20—C19—C18	121.86 (15)
C11—C8—C7	123.01 (15)	C21—C19—C18	119.68 (18)
C9—C8—C7	105.42 (14)	C19—C20—C19^i^	122.2 (2)
N2—C9—C8	111.78 (15)	C19—C20—H20	118.9
N2—C9—C10	118.33 (16)	C19^i^—C20—H20	118.9
C8—C9—C10	129.89 (16)	C22—C21—C19	120.0 (2)
C9—C10—H10A	109.5	C22—C21—H21	120.0
C9—C10—H10B	109.5	C19—C21—H21	120.0
H10A—C10—H10B	109.5	C21^i^—C22—C21	120.9 (3)
C9—C10—H10C	109.5	C21^i^—C22—H22	119.6
H10A—C10—H10C	109.5	C21—C22—H22	119.6
H10B—C10—H10C	109.5	C7—N1—N2	111.41 (13)
N3—C11—C8	119.28 (14)	C7—N1—C4	130.37 (15)
N3—C11—C12	118.49 (14)	N2—N1—C4	118.22 (14)
C8—C11—C12	122.23 (15)	C9—N2—N1	106.70 (14)
C13—C12—C17	119.14 (16)	C11—N3—C18	125.91 (14)
C13—C12—C11	121.05 (15)	C11—N3—H3A	117.0
C17—C12—C11	119.81 (16)	C18—N3—H3A	117.0
C12—C13—C14	119.96 (19)		
			
C6—C1—C2—C3	−0.4 (5)	C13—C14—C15—C16	1.8 (4)
C1—C2—C3—C4	0.1 (5)	C14—C15—C16—C17	−1.5 (4)
C2—C3—C4—C5	0.4 (4)	C13—C12—C17—C16	0.8 (3)
C2—C3—C4—N1	179.4 (2)	C11—C12—C17—C16	−178.25 (19)
C3—C4—C5—C6	−0.7 (4)	C15—C16—C17—C12	0.2 (4)
N1—C4—C5—C6	−179.7 (3)	N3—C18—C19—C20	−33.2 (2)
C2—C1—C6—C5	0.1 (6)	N3—C18—C19—C21	147.11 (18)
C4—C5—C6—C1	0.4 (5)	C21—C19—C20—C19^i^	−0.01 (13)
O1—C7—C8—C11	−0.4 (3)	C18—C19—C20—C19^i^	−179.73 (17)
N1—C7—C8—C11	179.42 (15)	C20—C19—C21—C22	0.0 (3)
O1—C7—C8—C9	−179.44 (17)	C18—C19—C21—C22	179.75 (15)
N1—C7—C8—C9	0.36 (18)	C19—C21—C22—C21^i^	−0.01 (13)
C11—C8—C9—N2	−178.64 (17)	O1—C7—N1—N2	178.94 (16)
C7—C8—C9—N2	0.3 (2)	C8—C7—N1—N2	−0.88 (18)
C11—C8—C9—C10	2.4 (3)	O1—C7—N1—C4	−0.6 (3)
C7—C8—C9—C10	−178.61 (19)	C8—C7—N1—C4	179.55 (18)
C9—C8—C11—N3	175.76 (17)	C5—C4—N1—C7	172.5 (2)
C7—C8—C11—N3	−3.0 (3)	C3—C4—N1—C7	−6.5 (3)
C9—C8—C11—C12	−3.1 (3)	C5—C4—N1—N2	−7.1 (3)
C7—C8—C11—C12	178.17 (15)	C3—C4—N1—N2	173.93 (18)
N3—C11—C12—C13	95.4 (2)	C8—C9—N2—N1	−0.8 (2)
C8—C11—C12—C13	−85.8 (2)	C10—C9—N2—N1	178.22 (16)
N3—C11—C12—C17	−85.6 (2)	C7—N1—N2—C9	1.09 (19)
C8—C11—C12—C17	93.2 (2)	C4—N1—N2—C9	−179.28 (16)
C17—C12—C13—C14	−0.5 (3)	C8—C11—N3—C18	175.89 (16)
C11—C12—C13—C14	178.51 (18)	C12—C11—N3—C18	−5.2 (3)
C12—C13—C14—C15	−0.8 (3)	C19—C18—N3—C11	−71.2 (2)

Symmetry codes: (i) −*x*, *y*, −*z*+1/2.

### Hydrogen-bond geometry (Å, °)

**Table:**

*D*—H···*A*	*D*—H	H···*A*	*D*···*A*	*D*—H···*A*
N3—H3A···O1	0.86	2.04	2.7353 (17)	138
